# From mRNA Expression of Drug Disposition Genes to In Vivo Assessment of CYP-Mediated Biotransformation during Zebrafish Embryonic and Larval Development

**DOI:** 10.3390/ijms19123976

**Published:** 2018-12-10

**Authors:** Evy Verbueken, Chloé Bars, Jonathan S. Ball, Jelena Periz-Stanacev, Waleed F. A. Marei, Anna Tochwin, Isabelle J. Gabriëls, Ellen D. G. Michiels, Evelyn Stinckens, Lucia Vergauwen, Dries Knapen, Chris J. Van Ginneken, Steven J. Van Cruchten

**Affiliations:** 1 Applied Veterinary Morphology, Department of Veterinary Sciences, University of Antwerp, 2610 Wilrijk, Antwerp, Belgium; evy.verbueken@uantwerpen.be (E.V.); chloe.bars@uantwerpen.be (C.B.); chris.vanginneken@uantwerpen.be (C.J.V.G.); 2 Biosciences, College of Life and Environmental Sciences, University of Exeter, EX4 4QD Exeter, UK; J.Ball@exeter.ac.uk (J.S.B.); A.Tochwin@exeter.ac.uk (A.T.); 3 Zebrafishlab, Veterinary Physiology and Biochemistry, Department of Veterinary Sciences, University of Antwerp, 2610 Wilrijk, Antwerp, Belgium; Jelena.perizstanacev@uantwerpen.be (J.P.-S.); Isabelle.gabriels@uantwerpen.be (I.J.G.); ellen.michiels@uantwerpen.be (E.D.G.M.); evelyn.stinckens@uantwerpen.be (E.S.); lucia.vergauwen@uantwerpen.be (L.V.); dries.knapen@uantwerpen.be (D.K.); 4 Gamete Research Centre, Veterinary Physiology and Biochemistry, Department of Veterinary Sciences, University of Antwerp, 2610 Wilrijk, Antwerp, Belgium; waleed.marei@uantwerpen.be; 5 Systemic Physiological and Ecotoxicological Research (SPHERE), Department of Biology, University of Antwerp, 2020 Antwerp, Belgium

**Keywords:** zebrafish, embryo, larva, development, cytochrome P450, phase II, drug transporter, biotransformation, activity, and expression

## Abstract

The zebrafish (*Danio rerio*) embryo is currently explored as an alternative for developmental toxicity testing. As maternal metabolism is lacking in this model, knowledge of the disposition of xenobiotics during zebrafish organogenesis is pivotal in order to correctly interpret the outcome of teratogenicity assays. Therefore, the aim of this study was to assess cytochrome P450 (CYP) activity in zebrafish embryos and larvae until 14 d post-fertilization (dpf) by using a non-specific CYP substrate, i.e., benzyloxy-methyl-resorufin (BOMR) and a CYP1-specific substrate, i.e., 7-ethoxyresorufin (ER). Moreover, the constitutive mRNA expression of *CYP1A*, *CYP1B1*, *CYP1C1*, *CYP1C2*, *CYP2K6*, *CYP3A65*, *CYP3C1*, phase II enzymes uridine diphosphate glucuronosyltransferase 1A1 (*UGT1A1*) and sulfotransferase 1st1 (*SULT1ST1*), and an ATP-binding cassette (ABC) drug transporter, i.e., *abcb4*, was assessed during zebrafish development until 32 dpf by means of quantitative PCR (qPCR). The present study showed that trancripts and/or the activity of these proteins involved in disposition of xenobiotics are generally low to undetectable before 72 h post-fertilization (hpf), which has to be taken into account in teratogenicity testing. Full capacity appears to be reached by the end of organogenesis (i.e., 120 hpf), although *CYP1*—except *CYP1A*—and *SULT1ST1* were shown to be already mature in early embryonic development.

## 1. Introduction

**Figure 1:**
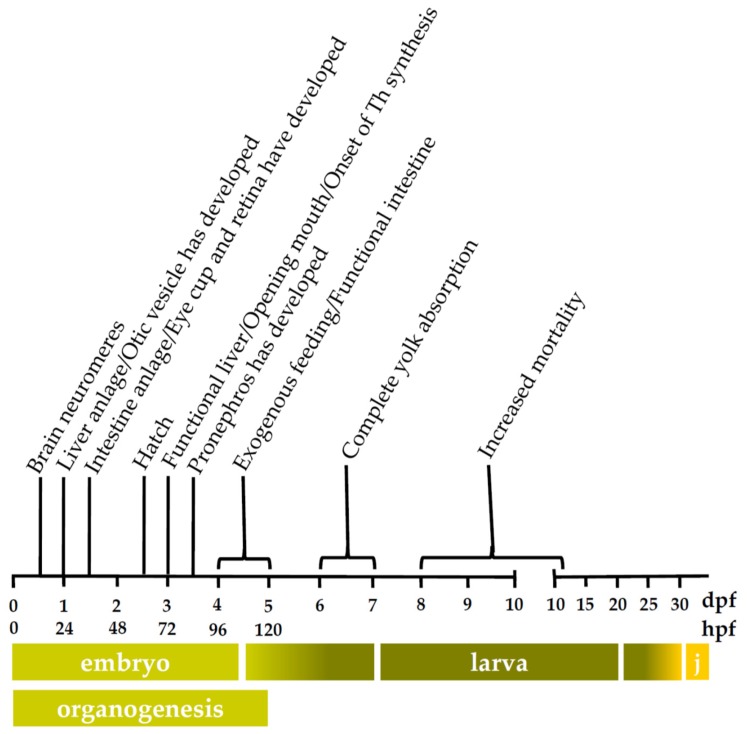
Timeline showing key events during zebrafish development, from fertilization to juvenile stages (j.) Color bars indicate the developmental phases with gradients representing embryo-larval and larval-juvenile transitions. The period of embryonic development includes pre-hatching stage and eleutheroembryo stage, i.e., the stage between hatching and onset of exogenous feeding [5,10]. The period of organogenesis, i.e., development of brain, heart, liver, intestine and pronephros, coincides with embryonic development. Embryo-larval transition implies the period between the onset of exogenous feeding and complete yolk absorption. Larval-juvenile transition reflects the period of metamorphosis in which the larval morphology is transformed into that of a juvenile (e.g., metamorphosis of the pigment pattern and fin morphology) [43,64]. Developmental stages of the organogenesis period are represented as h post-fertilization (hpf). Older developmental stages are shown as d post-fertilization (dpf). Th: thyroid hormone. The timeline is adapted from Vergauwen et al. [54] and based on Chang et al. [65], Drummond et al. [66], Field et al. [15], Kimmel et al. [6], Li et al. [67], Ng et al. [16], Ober et al. [17], Parichy et al. [64], Strähle et al. [5] and Wilson et al. [43].

The thalidomide tragedy in the late fifties and early sixties resulted in the obligatory use of a second, non-rodent, animal species in developmental toxicity studies. This second species, in most cases the rabbit, has proven to be very effective, as no cases of human birth defects that had not been flagged in animal studies have been reported ever since [1,2]. However, in view of cost and time effectiveness, and within the framework of the three Rs—Replacement, Reduction, and Refinement—described by Russell and Burch [3], the zebrafish (*Danio rerio*) embryo has been proposed as an alternative non-rodent animal model for developmental toxicity studies. Indeed, the zebrafish embryo is not considered to be a test animal until it reaches the stage of independent feeding, i.e., at 120 h post-fertilization (hpf) (Figure 1) [4,5]. Moreover, zebrafish are characterized by a rapid and ex utero embryonic development and embryos may be used in medium- or high-throughput screening because of their small size [6]. Hence, the zebrafish embryo developmental toxicity assay (ZEDTA) considers the physiological parameters of a whole organism together with the advantages of an in vitro model. Due to these benefits, several pharmaceutical companies and contract research organizations (CROs) have already adopted the ZEDTA as an early screening method to reduce the number of compounds that need to be tested in a mammalian model (reviewed in [7]). Further efforts are ongoing to explore regulatory acceptance of the ZEDTA in the drug development process [8,9]. At the same time, potential regulatory acceptance of the fish embryo acute toxicity test (FET) for chemical toxicity testing—according to the test guideline TG 236 of the Organization for Economic Co-operation and Development (OECD) [10]—is under consideration as an alternative for the fish acute toxicity test, i.e., TG 203 [11,12]. The FET (chemicals) uses exposure windows between 1.5 and 96 hpf [10], whereas the ZEDTA (pharmaceuticals) commonly uses exposure windows between 4 and 120 hpf to ensure that the entire zebrafish organogenesis period is covered [6,8,9,13,14].

The zebrafish liver and intestine, which are pivotal in the biotransformation of xenobiotics, become functional towards the end of the organogenesis period, i.e., around 96 hpf (Figure 1) [5,15,16,17]. Since zebrafish embryos develop ex utero, they are directly exposed to the parent compound in developmental toxicity assays. Only the chorion, which surrounds the embryo until 48–72 hpf [6], may serve as a barrier for certain compounds depending on their physicochemical properties [18]. Hence, zebrafish embryos depend on their intrinsic biotransformation capacity for the detoxification of xenobiotics and/or bioactivation of so-called proteratogens. In mammals, cytochrome P450 (CYP) families CYP1, CYP2 and CYP3 are involved in the oxidative (phase I) metabolism of xenobiotics as well as endogenous compounds such as steroids (reviewed in [19,20,21]). Goldstone and colleagues [22] suggested that also in adult zebrafish the CYP families 1–3 are involved in the biotransformation of xenobiotics. In humans and several laboratory mammals, CYP-mediated biotransformation capacity was shown to be immature during embryo-fetal development [23,24]. However, these embryos/fetuses can rely on maternal metabolism of the compound.

Based on the knowledge regarding the poor CYP-mediated drug metabolism in mammalian embryos/fetuses and the relatively late functional development of the zebrafish digestive system, i.e., liver and intestine, we hypothesize that the intrinsic CYP-mediated biotransformation capacity in zebrafish embryos is immature during early development. This implies that proteratogenic compounds may lead to false negative results in developmental toxicity studies if zebrafish embryos do not have the capacity to bioactivate those compounds. The hypothesis has been tested by an earlier in vitro study in which intrinsic CYP activity was assessed in microsomes—artificial subcellular fractions of endoplasmic reticulum containing CYPs—from whole zebrafish embryo homogenates at different time points between 5 and 120 hpf by means of a fluorogenic non-specific CYP substrate, i.e., benzyloxy-methyl-resorufin (BOMR) [25]. Biotransformation of BOMR into the fluorescent metabolite, i.e., resorufin, is a measure for the CYP activity in the microsomes. Since the liver and the intestine are the predominant sites for CYP-mediated drug metabolism (reviewed in [21]), intrinsic CYP activity was also assessed in liver microsomes prepared from adult female zebrafish as a reference for the embryos. In contrast to adults, zebrafish embryos showed no CYP-mediated metabolizing capacity in vitro during a major part of organogenesis, i.e., between 5 and 72 hpf, only poor CYP activity at 72 and 96 hpf and no CYP activity at 120 hpf [25]. Besides this in-house in vitro study, other research groups assessed (often after exposure to CYP inducers or inhibitors) CYP1 activity and, to a lesser extent, CYP3 activity during zebrafish organogenesis by using substrates that are specific for the respective CYP enzymes [26,27,28,29,30,31,32,33,34,35,36,37,38,39,40]. However, the overall results of these studies regarding the xenobiotic-metabolizing capacity of zebrafish embryos and larvae are contradictory, as some authors claim that zebrafish embryos show CYP-mediated biotransformation of xenobiotics [29,40], whereas others report that the extent of CYP-mediated biotransformation, e.g., metabolite concentrations, in zebrafish embryos and larvae is very low and unlikely to be relevant [27,39].

Therefore, the first aim of the present study was to further investigate the development of CYP activity in microsomes (in vitro) and in intact (in vivo) zebrafish embryos and larvae. As we noted a decrease in CYP activity at the end of zebrafish embryonic development, i.e., at 120 hpf, in a previous in vitro study [25], we wondered whether CYP-mediated biotransformation further matures, and if so, when. Therefore, we extended the developmental stages beyond the period of organogenesis (120 hpf) in the present study, i.e., including 9 and 14 d post-fertilization (dpf). At 96–120 hpf, exogenous feeding starts (Figure 1) and exposure to environmental compounds is expected to increase in a natural situation, which may activate the pregnane X receptor (PXR) or the aryl hydrocarbon receptor (AhR) that regulate CYP expression [41,42]. Hence, the onset of exogenous feeding may affect CYP activity in zebrafish larvae. Furthermore, larvae between 8 and 15 dpf often show increased mortality due to starvation in the period between complete yolk absorption and successful exogenous feeding (Figure 1), and this may affect CYP activity [5,43]. Besides an in vitro assessment, we also localized CYP-mediated biotransformation in intact zebrafish embryos and larvae, as organ-specific concentrations of the metabolite may be diluted when using microsomes prepared from whole organisms. Besides a non-specific CYP substrate, i.e., BOMR, we also included the CYP1-specific 7-ethoxyresorufin (ER) as a positive control substrate in the in vivo assay since the ethoxyresorufin-*o*-deethylase (EROD) assay is a well-established method in ecotoxicology to investigate the AhR-mediated induction of CYP1 enzymes by ubiquitous environmental contaminants such as 2,3,7,8-tetrachlorodibenzo-p-dioxin (TCDD) [26,44,45,46]. Furthermore, disposition of xenobiotics and endogenous compounds not only relies on phase I CYP-mediated biotransformation but also involves phase II reactions in which the parent compound or phase I metabolites are conjugated with a hydrophilic moiety, and cellular efflux by transporters in excretion organs, such as the liver, and barrier organs, such as the intestine (reviewed in [47]). In order to get a more complete view of the disposition in the zebrafish embryo and larvae, we decided to investigate the developmental mRNA expression of two major phase II enzymes in the zebrafish, i.e., sulfotransferase 1st1 (*SULT1ST1*) and uridine diphosphate glucuronosyltransferase 1A1 (*UGT1A1*) [48,49,50,51], and the ATP-binding cassette (ABC) drug transporter (*abcb4*). The latter was assessed since this transporter possesses similar multixenobiotic resistance (MXR) properties as the well-characterized mammalian ABCB1 transporter [52,53]. Since the same whole zebrafish body samples were used as in a study of Vergauwen et al. [54], the time window for the mRNA expression analysis was extended to 32 dpf to make the results comparable between both studies. As in literature, data on the ontogeny of *CYP1*, *CYP3* and, to a lesser extent, *CYP2* mRNA expression in zebrafish are limited to approximately 6 dpf [22,26,31,32,55,56,57,58,59,60,61,62,63], we decided to also include the constitutive mRNA expression of zebrafish *CYP1*, *CYP2*, and *CYP3* families at different time points between 1.5 hpf and 32 dpf in our assessment. Similar to the in vivo CYP activity study with BOMR, the mRNA expression of most CYP enzymes, phase II enzymes and P-glycoprotein reached maximum expression levels by the end of zebrafish organogenesis and remained stable throughout larval development. Hence, the present study showed general CYP-mediated biotransformation in zebrafish embryos towards the end of organogenesis, which needs to be considered with regards to the use of zebrafish embryos in ZEDTA and FET.

## 2. Results

### 2.1. In Vitro Study on Cytochrome P450 Activity in Zebrafish Embryos, Larvae and Adults

**Figure 2:**
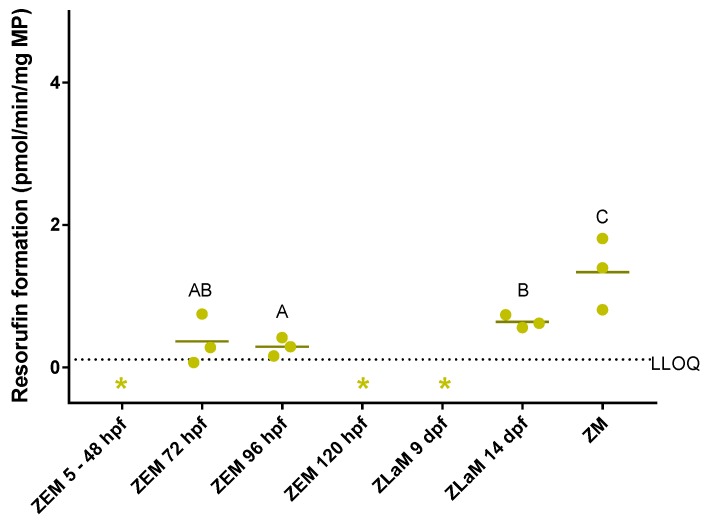
Resorufin formation (pmol/min/mg microsomal protein) by microsomes prepared from whole zebrafish embryos (ZEM) of between 5 and 120 h post-fertilization (hpf), microsomes prepared from whole zebrafish larvae (ZLaM) at 9 and 14 d post-fertilization and microsomes prepared from whole adult zebrafish (ZM) after incubation with benzyloxy-methyl-resorufin (BOMR). The dots are the reaction velocities for each biological replicate. Each dot represents the mean value of two, three and six technical replicates for ZEM, ZM and ZLaM, respectively. The horizontal solid line represents the mean reaction velocity of three biological replicates for each developmental stage. The horizontal dotted line represents the lower limit of quantification (LLOQ). The reaction velcoties for 5–48 hpf, 120 hpf and 9 dpf could not be calculated because of the negligible and non-linear metabolite concentrations (indicated by *). No statistically significant differences were detected between 72 hpf and 96 hpf and between 72 hpf and 14 dpf (*p* > 0.05). Statistically significant differences (*p* ≤ 0.05) between 96 hpf and 14 dpf and between ZM and the earlier stages, i.e., 72 hpf, 96 hpf and 14 dpf are indicated by different letters (A, B and C) (*p* = 0.050 for all comparisons).

CYP activity was assessed in microsomes prepared from whole zebrafish embryo homogenates (ZEM) of between 5 hpf and 120 hpf, in microsomes prepared from whole zebrafish larva homogenates (ZLaM) of 9 and 14 dpf and in microsomes prepared from whole adult zebrafish (ZM) by means of the benzyloxy-methyl-resorufin (BOMR) assay. The ZM were included in the assay as a reference for the embryos and larvae. The ZEM, which had been used in a former study [25], were included in the assay to show the development of CYP activity in zebrafish embryos and larvae. ZEM of 5, 24, 48 and 120 hpf and ZLaM of 9 dpf were not able to convert BOMR into the fluorescent metabolite, i.e., resorufin, as metabolite concentrations were negligible. Reaction velocities for ZEM of 72 and 96 hpf, for ZLaM of 14 dpf and for ZM were above the lower limit of quantification (LLOQ), i.e., mean reaction velocity of three biological replicates ± standard deviation (S.D.): 0.36 ± 0.35, 0.29 ± 0.13, 0.64 ± 0.09 and 1.34 ± 0.51 pmol/min/mg microsomal protein (MP) for the respective developmental stages (Figure 2). For the adult zebrafish liver microsomes (ZLM), which were included as a positive control, a reaction velocity of 9.65 ± 4.23 pmol/min/mg MP (mean value of six technical replicates for one biological replicate) was observed, which is in line with our previous study [25]. Furthermore, the BOMR assay with ZEM of between 5 and 120 hpf showed similar results as in a former study [25]. No statistically significant differences were detected between 72 hpf and 96 hpf (*p* = 0.827) and between 72 hpf and 14 dpf (*p* = 0.275). Statistically significant differences were detected between 96 hpf and 14 dpf and between ZM and the earlier stages, i.e., 72 hpf, 96 hpf and 14 dpf (*p* = 0.050 for all comparisons). ZLM and developmental stages with values below the LLOQ were not included in the statistical analysis.

### 2.2. In Vivo Study on Cytochrome P450 Activity in Zebrafish Embryos and Larvae

Since organ-specific concentrations of resorufin may be diluted when using microsomes prepared from whole zebrafish embryos and larvae, the aim of the in vivo study was to localize the biotransformation of BOMR in intact zebrafish embryos and larvae at 7, 26, 50, 74, 98, 122 hpf, 9 and 14 dpf. A quantitative and a qualitative analysis of resorufin formation in the trunk region of each embryo/larva was performed.

#### 2.2.1. Quantitative Analysis of Resorufin Formation

**Figure 3:**
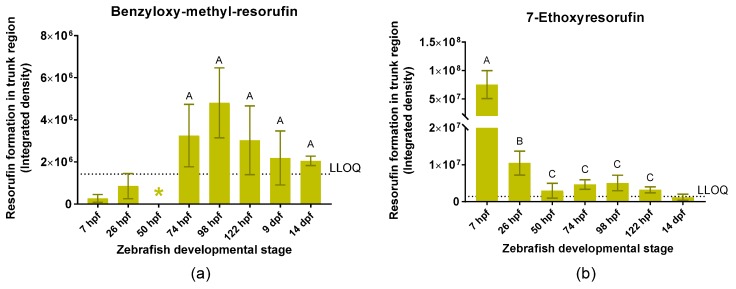
Integrated density of resorufin in the trunk region of intact zebrafish embryos and larvae at different time points during zebrafish development between 7 h post-fertilization (hpf) and 14 d post-fertilization (dpf) after incubation with benzyloxy-methyl-resorufin (BOMR) (**a**) and 7-ethoxyresorufin (ER) (**b**). At 7 hpf (**b**), integrated density of resorufin was determined in the whole embryo. Each bar represents the mean of three biological replicates ± standard deviation (S.D.). The horizontal dotted line represents the lower limit of quantification (LLOQ). In graph (**a**,**b**), developmental stages with values below the LLOQ were excluded from statistical analysis. In graph (**a**), no statistically significant differences (*p* > 0.05) were detected between the developmental stages that showed values above the LLOQ. The mean corrected integrated density value for 50 hpf was below zero (indicated by *). In graph (**b**), significant differences (*p* ≤ 0.05) between age groups are indicated by different letters (A, B and C): integrated density of resorufin was significantly higher at 7 and 26 hpf compared to the other developmental stages (*p* = 0.050 for all comparisons). Moreover, resorufin formation at 7 hpf was significantly higher than at 26 hpf (*p* = 0.050).

The BOMR substrate was not metabolized by zebrafish embryos of 7, 26, and 50 hpf, as the corrected integrated density of resorufin in the trunk region was below the LLOQ. However, embryos of 74, 98, and 122 hpf and larvae of 9 and 14 dpf were able to biotransform BOMR (integrated density of resorufin > LLOQ) (Figure 3a). No statistically significant differences were detected among the different age groups (*p* = 0.231).

Regarding the positive control, zebrafish larvae of 14 dpf were not able to biotransform 7-ethoxyresorufin (ER) as the corrected integrated density of resorufin in the trunk region was below the LLOQ. However, the ethoxyresorufin-*o*-deethylase (EROD) assay showed resorufin formation in embryos of 7, 26, 50, 74, 98, and 122 hpf (Figure 3b). Integrated density of resorufin was significantly higher at 7 and 26 hpf compared to the other developmental stages (*p* = 0.050 for all comparisons). Moreover, resorufin formation at 7 hpf was significantly higher than at 26 hpf (*p* = 0.050) (Figure 3b). The stage of 9 dpf was excluded from quantitative analysis since resorufin formation could not be localized due to a technical limitation, i.e., ventral position of the larvae.

#### 2.2.2. Qualtitative Analysis of Resorufin Formation

**Figure 4:**
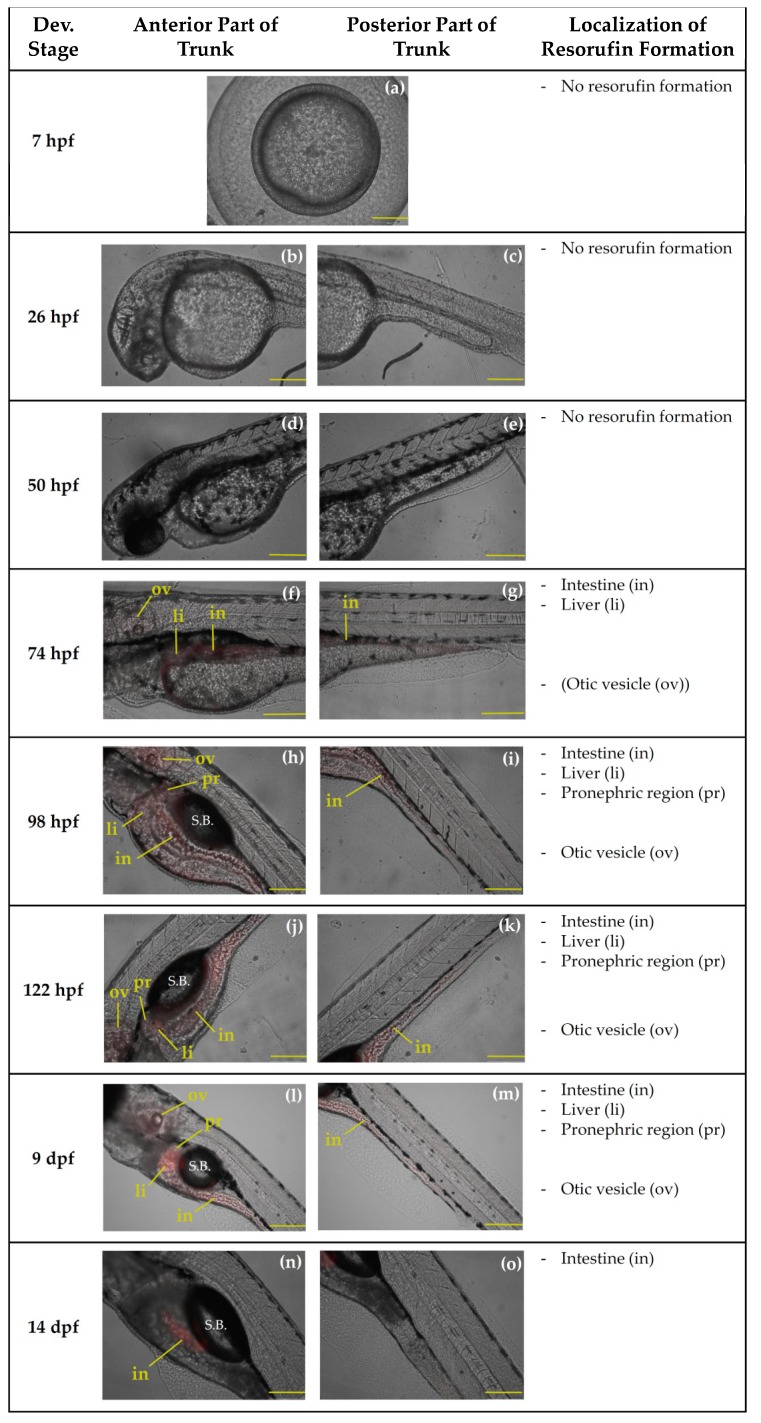
Localization of biotransformation of benzyloxy-methyl-resorufin (BOMR) in the trunk region of intact zebrafish embryos and larvae at 26 h post-fertilization (hpf) (**b**,**c**), 50 hpf (**d**,**e**), 74 hpf (**f**,**g**), 98 hpf (**h**,**i**), 122 hpf (**j**,**k**), 9 d post-fertilization (dpf) (**l**,**m**) and 14 dpf (**n**,**o**). At 7 hpf (**a**), qualitative analysis of resorufin formation was performed in the whole embryo. Pictures show one embryo/larva out of six used in the study, i.e., three biological replicates with two embryos/larvae per replicate, for each developmental stage. Figure 4**a** shows a vegetal pole view of the embryo. In Figure 4**b**–**o** lateral views of the anterior and posterior part of the trunk region are shown. The organs in which resorufin had been formed are indicated with a two-letter combination. Since the otic vesicle is part of the head region, resorufin formation in the respective organ is mentioned separately. S.B.: swim bladder. Scale bar: 200 µm; anterior left and dorsal top.

**Figure 5:**
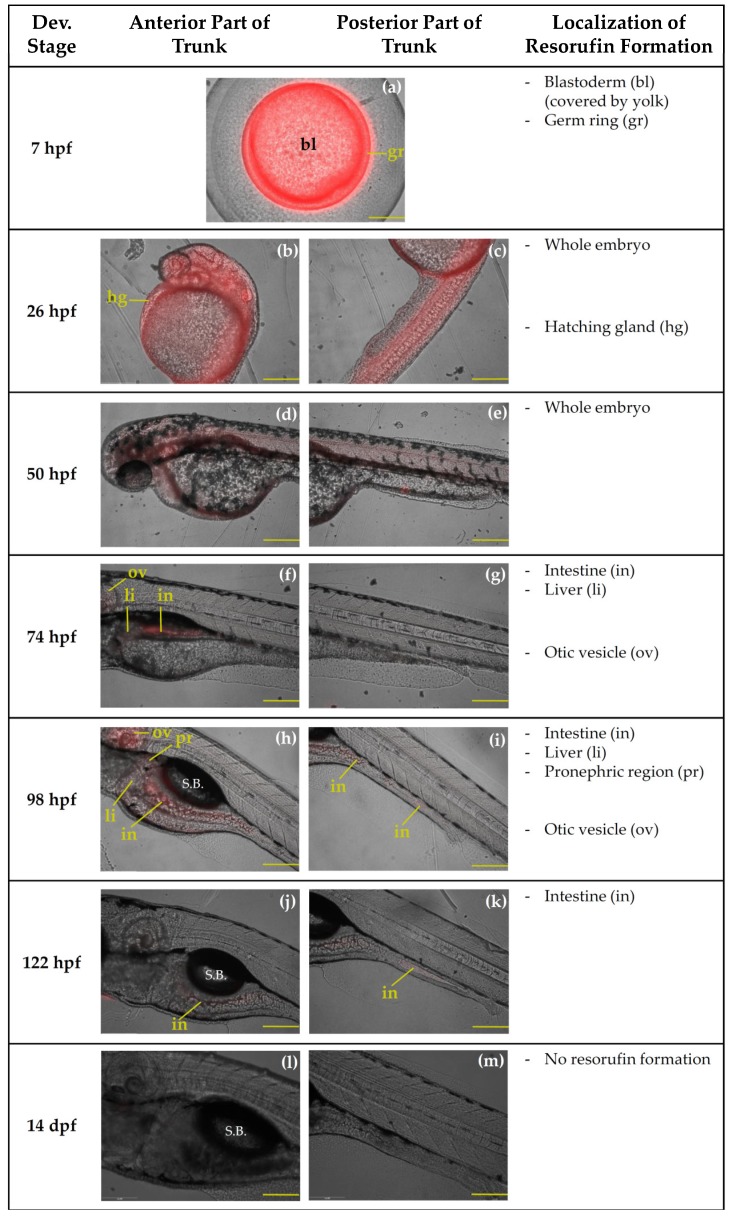
Localization of biotransformation of 7-ethoxyresorufin (ER) in the trunk region of intact zebrafish embryos and larvae at 26 h post-fertilization (hpf) (**b**,**c**), 50 hpf (**d**,**e**), 74 hpf (**f**,**g**), 98 hpf (**h**,**i**), 122 hpf (**j**,**k**) and 14 d post-fertilization (dpf) (**l**,**m**). At 7 hpf (**a**), qualitative analysis of resorufin formation was performed in the whole embryo. The stage of 9 dpf was excluded from the figure since resorufin formation could not be localized due to ventral position of the larvae. Pictures show one embryo/larva out of six used in the study, i.e., three biological replicates with two embryos/larvae per replicate, for each developmental stage. Figure (**a**) shows a vegetal pole view of the embryo. In Figure 5 (**b**–**m**) lateral views of the anterior and posterior part of the trunk region are shown. The organs in which resorufin had been formed are indicated with a two-letter combination. Since the hatching gland and otic vesicle do not belong to the trunk region, resorufin formation in the respective organs is mentioned separately. S.B.: swim bladder. Scale bar: 200 µm; (**b**,**c**): anterior top and dorsal right; (**d**–**m**): anterior left and dorsal top.

Biotransformation of BOMR was localized in the liver and intestine at 74, 98, 122 hpf and at 9 dpf (Figure 4f–m). At 14 dpf, resorufin formation was only detected in the intestine (Figure 4n,o). Furthermore, at this stage, there is food present and visible in the digestive tract. At 98, 122 hpf and at 9 dpf, the metabolite of BOMR was also observed in the pronephric region and, additionally, in the otic vesicle, which belongs to the head region (Figure 4h,j,l). A weak fluorescent signal was localized in the otic vesicle at 74 hpf (Figure 4f). Resorufin formation was not detected at 7, 26 and 50 hpf (Figure 4a–e). Similar to the in vivo BOMR assay, the positive control substrate, i.e., ER, was metabolized in the liver and intestine of zebrafish embryos of 74, 98, and 122 hpf (Figure 5f–k). In contrast to BOMR, biotransformation of ER was also observed at 7, 26 and 50 hpf (Figure 5a–e) with the strongest fluorescent signal in the germ ring at 7 hpf (Figure 5a). Since Figure 5a shows a vegetal pole view, the yolk covers the blastoderm resulting in a fluorescent signal of the blastoderm that is less intense. The embryo is entirely stained at 26 and 50 hpf (Figure 5b–e). Non-trunk-related structures such as the hatching gland and the otic vesicle showed resorufin formation at 26 hpf (Figure 5b) and at 50, 74, and 98 hpf, respectively (Figure 5d,f,h). The metabolite was not detected at 14 dpf (Figure 5l,m) and 9 dpf was excluded from the figure since resorufin formation could not be localized due to ventral position of the larvae.

### 2.3. mRNA Expression of Phase I and Phase II Enzymes and P–Glycoprotein

**Figure 6:**
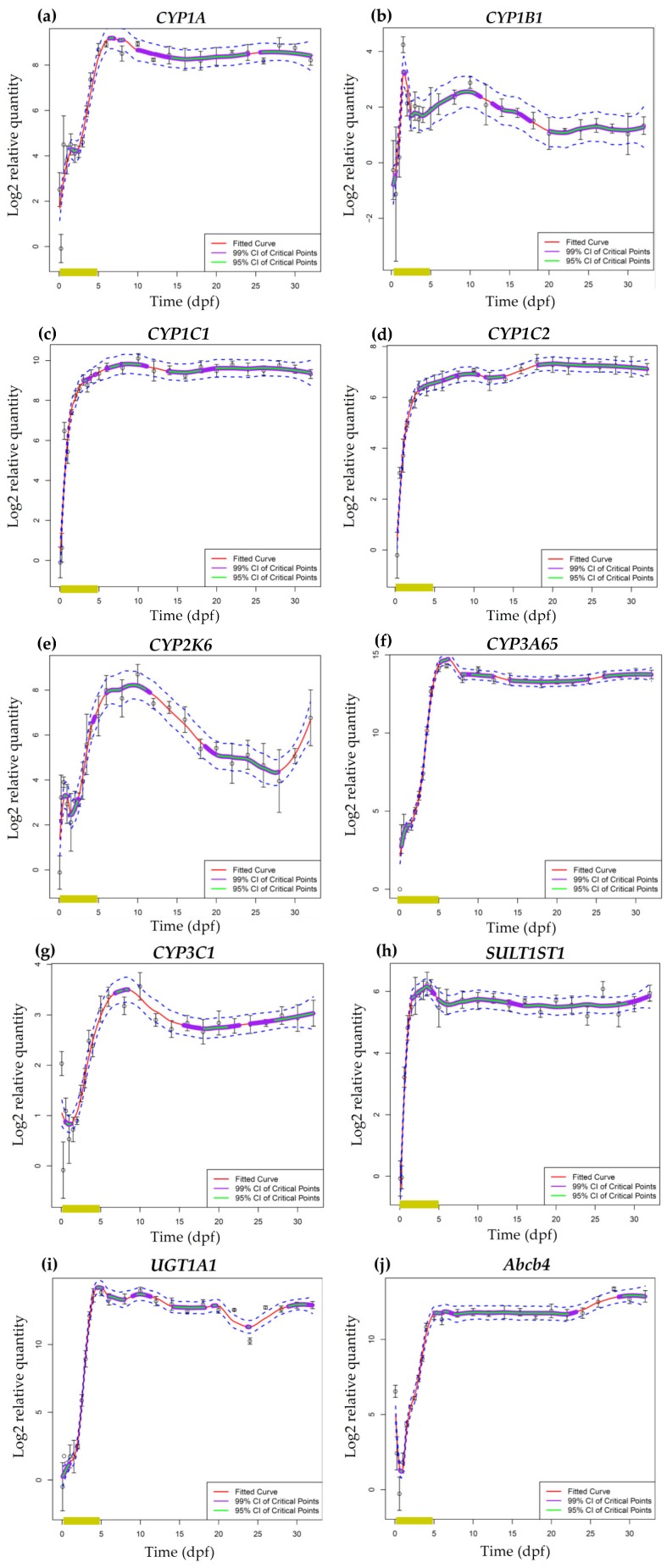
Relative quantities of cytochrome P450 (CYP) 1, 2 and 3 families from whole zebrafish bodies: (**a**) *CYP1A*, (**b**) *CYP1B1*, (**c**) *CYP1C1*, (**d**) *CYP1C2*, (**e**) *CYP2K6*, (**f**) *CYP3A65*, (**g**) *CYP3C1* and relative quantities of two phase II enzymes from whole zebrafish bodies, i.e., (**h**) sulfotransferase 1st1 (*SULT1ST1*) and (**i**) uridine diphosphate glucuronosyltransferase 1A1 (*UGT1A1*), and one P–glycoprotein, i.e., (**j**) ATP-binding cassette b4 (*abcb4*) transporter. The graphs show log2 relative quantities which were normalized for reference gene expression and expressed relative to the time point with the lowest expression. Data points represent mean ± S.D. of four replicate pools at each time point (days post-fertilization (dpf)). The red line indicates the loess fit of the gene target and the surrounding dashed blue line indicates the 95% confidence interval around the loess fit. The green and purple highlighted regions represent the 95% and 99% confidence intervals, respectively, of each critical point (minimum or maximum) of mRNA expression. The color bar between 0 and 5 dpf, i.e., between 0 and 120 h post-fertilization, indicates the period of zebrafish organogenesis.

The mRNA expression analysis was performed by means of a loess regression method in order to identify key inflection points, i.e., local maxima and minima, of transcriptional expression during zebrafish development. This method allowed us to identify statistically significant highs and lows in the expression profiles of phase I and phase II enzymes and P–glycoprotein (Figure 6). Data in Figure 6 are reported as log2 relative quantities—relative to the time point with the lowest expression—, which means that a log2 relative quantity of 2 for a particular time point corresponds to four times the expression of the time point with the lowest expression. Consequently, data should not be used for direct comparison of absolute expression levels among transcripts. Most transcripts only had low expression levels at the earliest time point, i.e., 1.5 hpf (Figure 6c–f,h,i). The *CYP1B1* transcript was not detected at 1.5 hpf (Figure 6b), whereas relatively high expression levels could be observed for *CYP1A*, *CYP3C1* and *abcb4* at this stage (Figure 6a,g,j). The high initial expression levels of *CYP1A* and *CYP3C1* were followed by a decline of mRNA expression between 1.5 and 6 hpf (Note that the regression does not capture this early decrease for CYP1A). Subsequently, transcript levels of *CYP1A* and *CYP3C1* increased from 14 hpf until 5–6 dpf after which both transcripts started to level out for the remaining developmental time points (Figure 6a,g). Within this period of increasing mRNA levels, *CYP1A* transcript levels remained stable between 14 hpf and 84 hpf (Figure 6a). The *Abcb4* transcript showed a similar expression pattern as for *CYP1A* but without the short period of stable mRNA expression during early embryonic development (Figure 6j). Regarding *CYP1C1*, *CYP1C2*, *CYP3A65*, *SULT1ST1* and *UGT1A1*, transcript levels showed a steep increase after the first time point, reached a maximum between 120 hpf and 10 dpf and remained stable for the remaining developmental time points (Figure 6c,d,f,h,i). A distinct pattern was observed for *CYP2K6* and *CYP1B1* since transcript levels reached a peak at 14 hpf and 36 hpf, respectively, followed by a decrease in expression levels until 48 hpf (Figure 6b,e). From 48 hpf onwards, *CYP1B1* transcripts started to level out with a slight fluctuation (Figure 6b), whereas *CYP2K6* mRNA levels started to increase until approximately 12 dpf followed by a decline until the end of the larval period. *CYP2K6* transcript levels tended to increase again by the beginning of the juvenile period (Figure 6e). These mRNA expression measurements have been performed in the same samples as those used in the study of Vergauwen et al. [54], where the ontogeny of thyroid related genes was studied. Hence, the current results can be directly related to the results of the previous study. The results of these studies can be directly compared via interactive graphs available online (http://zebrafishlab.be/ontogeny-explorer).

## 3. Discussion

### 3.1. Ontogeny of In Vitro and In Vivo Cytochrome P450 Activity in Zebrafish Embryos, Larvae and Adults

#### 3.1.1. In Vitro versus In Vivo

The results of the CYP activity assays support the hypothesis that the intrinsic CYP-mediated biotransformation capacity in zebrafish embryos is immature during early development although differences in CYP isoforms do occur. More specifically, biotransformation of the non-specific CYP substrate BOMR was above the LLOQ in intact embryos from 74 hpf onwards, i.e., towards the end of zebrafish organogenesis (Figure 1). Furthermore, these findings are in agreement with the present in vitro data that showed no BOMR biotransformation before 72 hpf in microsomes prepared from whole zebrafish embryo homogenates. This onset of CYP activity at 72 hpf coincides with vascularization of the liver, development of the intestinal epithelium and opening of the mouth (Figure 1). By 96 hpf, the liver has reached its adult configuration and the intestine has developed into an open-ended tube, which is reflected in CYP activity in the respective organs of intact embryos at 98 hpf [6,15,16,17,68]. This concurrence is not surprising as the liver and, to a lesser extent, the intestine are two major organs involved in mammalian CYP-mediated metabolism of xenobiotics [21,69]. However, there was a discordance between the in vitro and in vivo experiments with BOMR since in vitro CYP activity was low at 72 hpf, 96 hpf and 14 dpf, whereas in vivo CYP activity was clearly observed at 74 hpf, 98 hpf and 14 dpf and even at 122 hpf and 9 dpf. For the latter two stages, no CYP activity could be detected in vitro. The underestimation of CYP activity in the in vitro study is likely due to a dilution of the CYP enzymes, and consequently their activity, by other proteins in the microsomal fraction from the different tissues of the whole embryos/larvae. Indeed, the endoplasmic reticula of drug-metabolizing organs also contain other proteins such as flavin monooxygenases (FMOs) (phase I) and UDP glycosyltransferases (UGTs) (phase II) [70,71]. This dilution, and consequent underestimation of CYP activity, in whole organism homogenates was further substantiated by our in vitro CYP data in microsomes from whole adult zebrafish. The reaction velocities for BOMR biotransformation in microsomes prepared from whole adult zebrafish (ZM) and microsomes prepared from adult zebrafish livers (ZLM) were obviously different from each other, i.e., 1.34 pmol/min/mg MP versus 9.65 pmol/min/mg MP for the respective microsomes, which confirms our hypothesis regarding the dilution of CYP enzymes.

For a comprehensive discussion of the results of the in vitro study with BOMR in microsomes prepared from whole zebrafish embryos between 5 hpf and 120 hpf, we refer to Verbueken et al. [25].

#### 3.1.2. Benzyloxy-Methyl-Resorufin versus 7–Ethoxyresorufin

BOMR and ER biotransformation showed similar activity in the digestive system, i.e., between 74 and 122 hpf. The detection of resorufin formation in the digestive system at 74 hpf coincides with opening of the mouth around 72 hpf (Figure 1) [6]. At this stage, oral ingestion of xenobiotics complements uptake of compounds by the skin. Although Kais et al. [30] suggested that the detection of EROD activity in the intestine of zebrafish embryos is due to secretion of the metabolite from the liver via the bile, it should be noted that CYP families 1, 2 and 3 were shown to be expressed in the adult mammalian and zebrafish intestine [21,56,61,62,69,72]. Hence, the detection of resorufin formation in the intestine of zebrafish embryos from 74 hpf onwards may be attributed to biotransformation of the orally ingested BOMR or ER by intestinal CYP enzymes. Because of their role in mammalian drug metabolism, intestinal CYP enzymes are supposed to be involved in detoxification. In addition to the liver and intestine, resorufin formation was observed in the cranial pole of the early kidney, i.e., pronephros, of intact embryos and larvae of 98 hpf, 122 hpf and 9 dpf for BOMR and in embryos of 98 hpf for ER. The detection of CYP activity in the pronephros, which is involved in drug metabolism and elimination, follows the completion of pronephric nephron and filtration barrier development by 84 hpf [66]. Hence, the observation of CYP activity in the pronephros may be related to detoxification.

In contrast to BOMR, the present study showed that biotransformation of ER already occurred in the germ ring (blastoderm) at 7 hpf and in the whole embryo at 26 hpf and 50 hpf. This difference between both substrates may be explained by the fact that BOMR and ER have a different affinity for CYP1, 2 and 3 isoenzymes. Indeed, ER is known to be specifically metabolized by CYP1 isoenzymes, whereas BOMR was shown to be a non-specific CYP substrate according to a previous study with recombinant human CYP enzymes [25]. Although the current study does not provide a clear explanation for the presence of CYP1 activity in the early stages of zebrafish embryonic development, vertebrate CYP1 enzymes are known to play a role in embryonic development since CYP1B1 is involved in the synthesis of retinoic acid (RA), an endogenous signalling molecule which is essential in embryogenesis [73,74,75]. Furthermore, exogenous compounds are mainly taken up by the skin until opening of the zebrafish mouth and the onset of gill filament development, i.e., both around 72 hpf, [6]. Although not much is known about cutaneous CYPs in fish, the enzymes can be found in adult mammalian skin (reviewed in [21]) and RA was shown to be involved in mammalian embryonic skin development [76]. As such, CYP1 activity that was observed in the whole embryo at 26 and 50 hpf coincides with the period in which the substrate is taken up by the embryonic skin. In contrast to BOMR, EROD activity was not detected in larvae of 14 dpf (larvae of 9 dpf were not included in the assay because of difficulties with positioning). Regarding larvae of 9 and 14 dpf exposed to BOMR, the onset of exogenous feeding around 96–120 hpf and the increased mortality between 8 and 15 dpf did not affect the biotransformation of the substrate in the trunk region. However, the fluorescent signal in the digestive system of BOMR-exposed larvae of 14 dpf appeared to be less intense due to the presence of food in the digestive tract.

The trunk region was our main focus for the assessment of CYP activity since the major CYP-containing organs are located in this area. However, BOMR and ER were also metabolized in the otic vesicle—the zebrafish counterpart of the mammalian inner ear—at 74, 98, 122 hpf and 9 dpf for BOMR and at 74 and 98 hpf for ER. These stages do not coincide with the development of the respective organ as the otic vesicle and its corresponding otoliths have already been developed around 19 hpf and 22 hpf, respectively (Figure 1) [6]. However, in mammals, RA, and thus indirectly CYP enzymes, are suggested to be essential in embryonic development as well as in postnatal maintenance of the mammalian inner ear [74].

#### 3.1.3. Literature versus Current Study

According to literature, studies regarding the localization of CYP activity in intact zebrafish embryos mainly involve EROD assays [30,34], whereas to our knowledge, in vivo studies using a non-specific CYP substrate have not yet been described. Kais et al. [30] assessed EROD activities in intact zebrafish embryos of between 24 and 120 hpf, which are in line with the results of the present study. At 24 and 48 hpf, the authors reported biotransformation of ER in the whole embryo, the strongest fluorescent signal being located in the head region, i.e., brain, eyes and otic vesicle. However at 48 hpf, the fluorescent signal decreased compared to the previous stage, which is similar to our results for embryos of 50 hpf. From 72 hpf onwards, the authors reported EROD activity in the digestive system, which slightly increased until 96 hpf and remained stable at 120 hpf [30]. A study from Otte et al. [34] included zebrafish embryos of 8 hpf that showed biotransformation of ER in the blastoderm, similar to the youngest embryos in the current study. The authors also localized EROD activity in zebrafish embryos of 32, 56, 80, 104, and 128 hpf, in similar organs as in the current study for the corresponding stages, i.e., 26, 50, 74, 98, and 122 hpf respectively. However, Otte and colleagues [34] were able to show a more detailed localization of CYP1 activity e.g., in myotomes, pronephric duct, vessels, organ primordia, etc. These anatomical structures were visualized by a confocal laser scanning microscope (CLSM) which makes high resolution images possible due to the process of optical sectioning [34]. Since in the present study and in the one from Kais et al. [30], an epifluorescence microscope had been used, organs like the pronephric duct and vessels could not be distinguished from the surrounding structures.

Because of the similarities with the results described in literature, we may conclude that ER is suited as a positive control in CYP activity assays with intact zebrafish embryos/larvae.

### 3.2. Ontogeny of Cytochrome P450 mRNA Expression in Zebrafish Embryos and Larvae

As CYP transcript levels have been investigated in zebrafish embryos by other groups before, we will focus the discussion mainly on the later stages.

#### 3.2.1. *Cytochrome P450* mRNA Expression during Zebrafish Organogenesis

For all *CYP* enzymes that were investigated, mRNA expression levels increased during the organogenesis period. Moreover, the increase in *CYP1* transcript levels before 72 hpf was concomitant with the results of the EROD activity assay.

In the current study, the relatively high expression levels for *CYP1A* and *CYP3C1* at 1.5 hpf suggest maternal transfer of the respective mRNA transcripts since the zebrafish zygotic genome becomes gradually activated in the blastula period throughout a window of approximately two hours, starting at cell cycle 10 (around 2.75 hpf according to Kimmel et al. [6]) (reviewed in [77]). The maternal mRNA transcripts are produced during oogenesis and are present in the egg at fertilization. They are considered to be essential for the development of the earliest embryonic stages (reviewed in [77]). Moreover, a recent study compared fertilized eggs of 1.5 hpf with unfertilized eggs for zebrafish thyroid-related transcript levels and was not able to detect differences between both conditions, which confirms that the detection of transcript levels at 1.5 hpf is due to maternal transfer [54].

No maternal transfer was detected for *CYP1B1*, *CYP1C1*, *CYP1C2*, *CYP2K6*, and *CYP3A65*. However, mRNA levels increased immediately after activation of the embryonic genome (around 6 hpf). Transcript levels of *CYP1C1*, *CYP1C2*, and *CYP3A65* showed a steep increase throughout the organogenesis, whereas a distinct pattern was observed for *CYP1B1* and *CYP2K6* mRNA levels. Indeed, *CYP1B1* transcripts peaked at 36 hpf, followed by a decline until 48 hpf after which mRNA levels started to level out for the remaining developmental time points. This peak, which was also detected by Goldstone et al. [22] at the same time point, coincides with the development of the eye cup and retina (Figure 1) [6,67]. Moreover, Yin and colleagues [78] already reported basal *CYP1B1* mRNA expression in ocular cells of zebrafish at 24 hpf after which transcription levels peaked between 30 and 48 hpf. In addition to the eye, *CYP1B1* mRNA levels were detected in the zebrafish brain at 36 and 48 hpf by whole-mount in situ hybridization [78]. Also in human fetuses, *CYP1B1* mRNA was abundantly expressed in the brain [79]. Regarding *CYP2K6*, transcript levels in the current study peaked at 14 hpf followed by a decrease until 48 hpf. In contrast to *CYP1B1, CYP2K6* mRNA levels started to increase again after hatching until 10 dpf. In a study of Wang-Buhler et al. [63], *CYP2K6* transcripts were expressed in liver and ovary of adult zebrafish. However, the presence of *CYP2K6* transcripts in adult zebrafish liver and ovary cannot explain the high transcript level at 14 hpf in the current study since these organs develop later. Yet, the early *CYP2K6* mRNA peak coincides with the development of the brain neuromeres (Figure 1) around 16 hpf and with the onset of heart development around 16–19 hpf [6,80]. With regards to *CYP1C1* and *CYP1C2*, Jönsson et al. [72] described an increase in basal mRNA levels from 8 to 96 hpf and from 8 to 72 hpf for the respective enzymes, which is similar to the present study. However, the same authors showed fluctuating *CYP1C1* and *CYP1C2* mRNA levels between 96 hpf and 7 dpf, whereas transcript levels remained stable in the current study [72]. Regarding *CYP3A65*, the present study and the one from Tseng et al. [62] both reported increasing mRNA levels throughout the organogenesis period. Moreover, *CYP3A65* transcripts were detected in the liver at 72 hpf by whole-mount in situ hybridization and subsequently in liver and intestine at 84, 96, and 120 hpf [62]. In contrast to the current study, maternal *CYP3A65* transcripts were observed at 3 hpf by Goldstone et al. [22] and a study of Glisic et al. [81] showed low *CYP3A65* mRNA expression levels until 96 hpf followed by a peak at 120 hpf.

We can conclude that, with regards to the zebrafish organogenesis period, the results of *CYP* mRNA expression analysis are in accordance with the majority of studies described in literature.

#### 3.2.2. *Cytochrome P450* mRNA Expression during Zebrafish Larval Development

Except for *CYP1B1*, all *CYP* transcripts that were investigated reached maximum expression levels during embryo-larval transition, i.e., between 4 and 7 dpf (Figure 1), which comprises the period between the onset of exogenous feeding and complete yolk absorption. Exogenous feeding implies increased exposure to environmental compounds, which may cause a slight induction of *CYP* mRNA expression due to PXR or AhR activation [41,42]. However, *CYP1C1* and *CYP1C2* transcripts reached high expression levels already around 72 hpf, which coincides with the opening of the mouth. This implies an increased exposure of the zebrafish embryo to exogenous compounds that are present in the fish medium, which might result in an induction of the respective *CYP* enzymes.

After reaching maximum mRNA levels during the embryo-larval transition, *CYP1A*, *CYP1C1*, *CYP1C2*, and *CYP3A65* transcript levels remained stable throughout the larval period, whereas mRNA levels of *CYP1B1*, *CYP2K6*, and *CYP3C1* fluctuated to some extent. The decline of transcript levels around 10 dpf that was observed for *CYP1B1*, *CYP2K6*, and *CYP3C1* coincides with the period of increased mortality due to starvation (Figure 1). However, the correlation between both observations remains unclear. A more plausible explanation for the fluctuating *CYP* transcript levels during the larval period might be changes in the environment such as feeding regimen and stocking density. In a study of Wang-Buhler et al. [63], *CYP2K6* transcript levels were detected in liver and ovary of adult zebrafish. Hence, the decline in *CYP2K6* mRNA levels throughout the larval period might be explained by the decrease in relative liver size in proportion to the increasing body mass. The subsequent increase in *CYP2K6* transcript levels at the larval-juvenile transition period may be attributed to the onset of gonad development around 30 dpf [82]. Regarding *CYP1A*, *CYP1C1*, *CYP1C2*, and *CYP3A65*, mRNA expression remained constant during the larval period despite the growth burst between 9 and 51 dpf [83] and the corresponding decline of relative organ size and organ-specific *CYP* mRNA expression. This might be due to a shift or increase of organ-specific CYP mRNA expression, which results in constant transcript levels throughout the whole larval body. In the study of Jönsson et al. [72], transcript levels of the *CYP1* family were assessed until 57 dpf, which is still within the juvenile period of between 30 and 90 dpf. In contrast to the present study, Jönsson et al. [72] showed fluctuating *CYP1A* and *CYP1C1* mRNA levels throughout the larval period. Regarding larval *CYP1B1* and *CYP1C2* mRNA expression, the results are in accordance with the present study. To our knowledge, no other *CYP* mRNA expression studies covering the whole zebrafish larval period have been performed.

### 3.3. Ontogeny of mRNA Expression of Two Phase II enzymes and a P–glycoprotein in Zebrafish Embryos and Larvae

The biotransformation of xenobiotics and endogenous compounds implies phase II reactions in which the parent compound or phase I metabolites are conjugated with a hydrophilic moiety to enhance their water solubility and elimination from the body. In the present study, the embryonic and larval development of the constitutive mRNA expression of two major phase II enzymes, i.e., sulfotransferase 1st1 (*SULT1ST1*) and uridine diphosphate glucuronosyltransferase 1A1 (*UGT1A1*) was assessed in zebrafish. In mammals, UGT enzymes are located predominantly in the endoplasmic reticulum of liver, intestine, kidney, lungs, skin, brain and spleen, whereas SULT enzymes are primarily located in the cytosol of liver, intestine, kidney, lung, platelets and brain. Conjugation reactions comprise glucuronidation and sulfonation by UGT and SULT enzymes, respectively (reviewed by [84]). Zebrafish *UGT1A* was first identified by Huang and Wu [50] and is expressed in liver and intestine and, to a lesser extent, in brain and testis of adult zebrafish [48]. In the current study, *UGT1A1* transcripts reached maximum expression levels during embryo-larval transition (Figure 1) after which mRNA levels levelled off throughout the larval period. Since embryo-larval transition coincides with the onset of exogenous feeding and since *UGT1A* is supposed to be regulated through the AhR pathway [48], we assume that the increased exposure to environmental compounds induced *UGT1A1* mRNA expression due to AhR activation. Christen and colleagues [48] assessed *UGT1A* mRNA expression between 24 and 120 hpf, and showed an increase in transcript levels between 48 and 120 hpf, which is in line with the present study. However, in contrast to the current study, *UGT1A* mRNA levels at 24 hpf were higher than at 48 hpf [48]. Zebrafish *SULT1ST1*, which was first identified by Liu et al. [51], showed maximum transcript levels already around 72 hpf, which coincides with the first observation of thyroid hormone synthesis (Figure 1) [65]. Moreover, zebrafish SULT1ST1 enzymes are involved in the sulfonation of endogenous thyroid hormones [85], which might explain the maximum *SULT1ST1* mRNA levels around 72 hpf. After reaching a maximum in the current study, *SULT1ST1* transcript levels remained stable throughout the larval period. In contrast to the present study, Liu et al. [51] showed low levels of *SULT1ST1* mRNA expression in unfertilized eggs and in embryos immediately after fertilization, suggesting maternal transfer of the transcript.

Besides phase I and phase II enzymes, the bioavailability of xenobiotics also depends on the presence of ATP-binding cassette (ABC) drug transporters that protect cells against a wide range of xenobiotics. Zebrafish *abcb4*, which was first described by Fischer et al. [53], possesses similar functional properties as the mammalian ABCB1 transporter. The present study showed maternal transfer of *abcb4* transcripts, which suggests that Abcb4 is essential for the protection of the early embryo against environmental compounds. Subsequently, *abcb4* transcript levels declined at 6 and 14 hpf followed by an increase around 24 hpf. *Abcb4* transcripts rose until 120 hpf followed by stable mRNA levels throughout the larval period. The temporal expression profile of *abcb4* is in line with the study of Fischer et al. [53] in which transcript expression was assessed until 48 hpf.

In the literature, not much is known about the activity of phase II enzymes and P-glycoproteins during zebrafish development, nor about their possible role in embryogenesis.

## 4. Materials and Methods

### 4.1. In Vitro Study on Cytochrome P450 Activity in Zebrafish Embryos, Larvae and Adults

#### 4.1.1. Fish Maintenance and Breeding

- Fish Maintenance and Breeding: Zebrafish Embryos

For a description of fish maintenance and breeding with regards to zebrafish embryos of between 5 hpf and 120 hpf, we refer to Verbueken et al. [25].

- Fish Maintenance and Breeding: Zebrafish Larvae

Adult zebrafish (*Danio rerio*, wild-type AB zebrafish line obtained from European Zebrafish Resource Center at Karlsruhe Institute of Technology, Germany), which were used for spawning, were housed in enriched aquaria of 40 L at a density of ≤5 fish/L and at an automated 14/10 h light/dark cycle. Fish were kept in fish medium, i.e., reverse osmosis water (Environmental Water Systems Ltd., Cheddar, UK) to which commercial sea salts (Tropic Marin^®^ Sea Salt, Wartenberg, Germany) and NaHCO_3_ (Analar Normapur^®^, VWR International, Leicestershire, UK) were added in order to obtain pH and conductivity values of 8 and 350 µS/cm, respectively. The water temperature was set to 28 °C and levels of ammonia, nitrite and nitrate were kept below the permissible limits, i.e., NH_3_ < 0.25 mg/L, NO_2_^−^ < 0.3 mg/L and NO_3_^−^ < 20 mg/L. Adult fish were fed freshly harvested *Artemia* nauplii (ZM Premium Grade Artemia, Zebrafish Management Ltd., Winchester, UK) and tropical flake food (TetraMin, Tetra^®^, Melle, Germany) twice daily.

Embryos were generated by a group spawning method as detailed in Gustafson et al. [9]. Briefly, eggs were collected within 45 min after spawning and incubated in fish medium at 28 ± 1 °C for 1–2 h. Subsequently, embryos were treated against fungal infection using a dilute Chloramine T bleaching solution for 60 s with gentle periodic agitation. Following bleaching, the embryos were washed twice in fish medium with constant agitation, then transferred into a Petri dish (50 embryos per dish) containing aerated fish medium at 28 ± 1 °C. Embryos were staged for development according to methods that have been previously described by Kimmel et al. [6]. At 120 hpf embryos were transferred to a crystallizing dish container (0.5 embryo/mL) and raised until 9 or 14 dpf. Fish medium was partially (25%) renewed daily and larvae were fed with dry feed three times a day according to the following scheme: ZM-000 (Zebrafish Management Ltd.) for 5–8 dpf, a mix of ZM-000 and ZM-100 (Zebrafish Management Ltd.) for 9–10 dpf and ZM-100 for 11–14 dpf. Larvae were euthanized by an overdose of tricaine methane sulfonate (MS222; 2 mg/mL) (Sigma-Aldrich, St. Louis, MO, USA) when they reached the desired developmental stage, i.e., 9 or 14 dpf. The terminated larvae were snap-frozen with as less fish medium as possible in liquid nitrogen and stored at −80 °C until processing. The animal protocols in this study were evaluated and approved by the UK Home Office regulations and the local ethic committee for the use of animals in scientific procedures (project number 17-002. 70/98992; August 2016; Exeter University Animal Welfare and Ethics Review Body). In this research paper, developmental stages of the organogenesis period are represented as h post-fertilization (hpf), similar to the time unit used in developmental toxicity studies. Older developmental stages are shown as d post-fertilization (dpf).

- Fish Maintenance: Adult Zebrafish

Zebrafish (*Danio rerio*, in-house wild-type AB zebrafish line) that were used for the isolation of microsomes from whole adult homogenates were housed in an aquarium of 400 L at a density of ≤5 fish/L and at an automated 14/10 h light/dark cycle. Fish were kept in tap water at 28 °C and levels of ammonia, nitrite and nitrate were kept below the permissible limits, i.e., NH_3_ < 0.25 mg/L, NO_2_^−^ < 0.3 mg/L and NO_3_^−^ < 20 mg/L. The fish were fed three times a day with granulated food (Biogran medium, Prodac International, Cittadella, Italy).

Adult zebrafish were euthanized by rapid cooling in ice water at 2–4 °C to (no physical contact with ice) followed by decapitation and destruction of the brain [86]. Subsequently, the gall bladder was removed from the body since bile acids are detrimental for the CYP activity in the sample. The terminated fish were snap-frozen in liquid nitrogen and stored at −80 °C until processing. Since the adult zebrafish were used for breeding, no ethical approvement was needed for the preparation of microsomes from whole adult homogenates.

#### 4.1.2. Tissue Collection and Isolation of Microsomes

- Isolation of Microsomes from Whole Zebrafish Embryos

For a description of tissue collection and isolation of microsomes with regards to zebrafish embryos of between 5 hpf and 120 hpf, we refer to Verbueken et al. [25].

- Isolation of Microsomes from Whole Zebrafish Larvae

Two biological replicates of approximately 500 larvae and three biological replicates of approximately 700 larvae were used for microsomal protein preparation of 9 and 14 dpf, respectively. The microsomes prepared from whole zebrafish larvae were isolated according to the same protocol as described by Verbueken et al. [25]. Briefly, homogenized zebrafish larvae were centrifuged at 12,000× *g* which resulted in a supernatant that contains the S9-fraction. The resulting supernatant was then subjected to two ultracentrifugation steps at 100,000× *g* to render a microsomal pellet. Finally, the microsomal protein concentration was determined by means of the microplate procedure of the Pierce^TM^ BCA Protein Assay Kit with bovine serum albumin as a standard (Thermo Fisher Scientific, Waltham, MA, USA).

- Isolation of Microsomes from Whole Adult Zebrafish

Three biological replicates, each consisting of seven adult zebrafish of mixed genders, were used for the preparation of microsomes from whole adult zebrafish homogenates. Frozen adult fish were homogenized by crushing them into a fine powder. After the addition of homogenization buffer (10 mM KPO_4_ buffer containing 1.15% KCl, 1 mM ethylenediaminetetraacetic acid (EDTA) and 1 unit of Halt^TM^ Protease Inhibitor Single-Use Cocktail per 10 mL buffer (the latter two were purchased from Thermo Fisher Scientific, Waltham, MA, USA) at pH 7.4) to the crushed tissue, an additional homogenization step was performed by means of a Polytron^®^ System PT 1200 E (Kinematica Inc., Bohemia, NY, USA). As a final homogenization step, samples were subjected to ultrasonication for (5 × 5) s with intervals of 10 s and an amplitude of 75% using an Ultrasonic Processor VCX 130 (Sonics & Materials Inc., Newton, CT, USA). The microsomes were isolated from the whole adult zebrafish homogenates according to the same protocol as described for the zebrafish embryos and larvae in the current study [25]. Finally, the microsomal protein concentration was determined by means of the microplate procedure of the Pierce^TM^ BCA Protein Assay Kit with bovine serum albumin as a standard (Thermo Fisher Scientific, Waltham, MA, USA).

#### 4.1.3. Benzyloxy-Methyl-Resorufin Assay in Microsomes Prepared from Whole Zebrafish Embryos, Larvae and Adults

In a previous study [25], benzyloxy-methyl-resorufin (Vivid^®^ BOMR Substrate, P2865, Thermo Fisher Scientific) was shown to be a fluorogenic non-specific CYP substrate in the zebrafish. Biotransformation of BOMR into resorufin by zebrafish microsomes is a measure for the CYP activity in the respective microsomes. In this study, BOMR was used to assess CYP activity in microsomes prepared from (1) whole zebrafish embryo homogenates (ZEM) of 5, 24, 48, 72, 96, and 120 hpf, (2) whole zebrafish larva homogenates (ZLaM) of 9 and 14 dpf and (3) whole adult zebrafish homogenates (ZM). A microsomal protein concentration of 200 µg/mL and a BOMR substrate concentration of 1.2 µM were used in the activity assay as both values were situated within the linear part of the reaction curve in an optimization study with adult female zebrafish liver microsomes (ZLM) [25]. Insect Cell Control Supersomes™ (456201, Corning Inc., Corning, NY, USA), lacking CYP enzymes, were chosen as negative control. ZLM (Batch 3, prepared from the livers of 10 adult zebrafish) that had shown positive results in a previous study [25] were used as a positive control. Hence, only one biological replicate of ZLM was used in the current assay. The ZEM (Batch 1–3), which had already been used in a previous study [25] were included in the assay to assess CYP activity throughout zebrafish development. Positive and negative controls and ZEM were subjected to the same protein and substrate concentration as for ZLaM and ZM. The CYP activity assays were performed in non-binding black polystyrene 96-well microplates with flat bottom and chimney wells (655900, Greiner Bio-One International GmbH, Kremsmünster, Austria). A total incubation volume of 100 µL/well was used. The microsomal reaction was initiated in each well by the addition of substrate solution containing 1.2 µM BOMR, 0.1 mM NADP^+^ (Vivid^®^ NADP+, P2879, Thermo Fisher Scientific), 3.33 mM glucose-6-phosphate, 0.3 U/mL glucose-6-phosphate dehydrogenase (Vivid^®^ Regeneration System, P2878, Thermo Fisher Scientific) and 100 mM KPO4 buffer (pH 7.4) (Corning, Discovery Labware Inc., Woburn, MA, USA) to the microsomal solution containing 20 µg/100 µL microsomal protein and 100 mM KPO4 buffer (pH 7.4) under light-protected conditions. Subsequently, fluorescence was measured for 72 min with 150-s intervals using a Tecan Infinite^®^ 200 PRO microplate reader (Tecan Group Ltd., Männedorf, Switzerland) at *λ*_ex_ 550 nm and *λ*_em_ 590 nm. During measurement, the temperature was kept at 28 °C which is within the zebrafish’s optimal water temperature range of 26–28.5 °C [87]. The concentration of resorufin (nM)—a metabolite of BOMR—produced at each time point was determined from a standard curve that had been established by using the pure fluorescent metabolite (Vivid^®^ Red Fluorescent Standard, P2874, Thermo Fisher Scientific). The average values of the negative control were subtracted from the individual result values obtained for ZLaM, ZEM, ZM, and ZLM. Reaction velocities were calculated in units of picomoles of resorufin formed per minute per milligram of microsomal protein (pmol/min/mg MP). For each batch of ZLaM, six technical replicates of the activity assay were performed and for each batch of ZM, three technical replicates of the activity assay were performed (Table 1). Only two technical replicates were included for ZEM of 5–120 hpf as for the latter, CYP activity had already been assessed [25].

#### 4.1.4. Mathematical and Statistical Analyses

The reaction velocities were calculated within the linear part of the reaction curve. The lower limit of detection (LLOD) was 0.07 pmol/min/mg MP, and lower limit of quantification (LLOQ) was 0.11 pmol/min/mg MP. These limits were determined based on the mean and standard deviation of the negative control values as described by Şengül [88]. Calculation of reaction velocities and detection and quantification limits was performed in Microsoft Excel^®^ 2016 (Microsoft Corporation, Redmond, WA, USA). The results from the CYP activity assays with BOMR that showed reaction velocities above the LLOQ were statistically analyzed using IBM SPSS Statistics (version 25; IBM, Armonk, NY, USA). A nonparametric Levene’s test was used to test homogeneity of variances for ZEM of 72 hpf and 96 hpf, ZLaM of 14 dpf and ZM. Subsequently, the results for these age groups were subjected to a Kruskal-Wallis test, followed by pairwise comparisons (Mann-Whitney test) to detect differences between the groups. Differences were considered statistically significant when *p* ≤ 0.05.

### 4.2. In Vivo Study on Cytochrome P450 Activity in Zebrafish Embryos and Larvae

#### 4.2.1. Fish Maintenance and Breeding

Adult zebrafish (*Danio rerio*, in-house wild-type zebrafish line) were housed in a ZebTEC zebrafish housing system (Tecniplast, Buguggiate, Italy) at an automated 14/10 h light/dark cycle, at 28 ± 0.2 °C. Fish were kept in fish medium, i.e., reverse osmosis water (Werner, Leverkusen, Germany) to which commercial sea salts (Instant Ocean^®^ Sea Salt, Blacksburg, VA, USA) and NaHCO_3_ (Analar Normapur^®^) were added in order to obtain pH and conductivity values of 7.5 and 500 µS/cm, respectively. Around 35% of the circulating water was renewed daily to keep levels of ammonia, nitrite and nitrate below the permissible limits, i.e., NH_3_ < 0.25 mg/L, NO_2_^−^ < 0.3 mg/L and NO_3_^−^ < 20 mg/L. Adult fish were fed three times a day: twice with 0.5% of their mean wet weight of granulated food (Biogran medium, Prodac International, Cittadella, Italy) and once with thawed food: alternating *Artemia* nauplii, *Daphnia*, Chaoboridae larvae and Chironomidae larvae (Aquaria Antwerp bvba, Aartselaar, Belgium).

For the collection of zebrafish embryos, two female fish and one male fish were transferred to a breeding tank and separated from each other the evening before mating. The next morning, the divider was removed when the lights turned on. After 30–40 min, eggs were collected from multiple spawning groups and distributed into plastic beakers with an initial density of 0.4 embryo/mL. Zebrafish embryos were raised in fish medium that had the same composition as the water in the ZebTEC housing system and under the same environmental conditions of light and temperature as for the adults. Dead embryos were removed daily and the fish medium was renewed every two days until 120 hpf. From 120 hpf until 9 dpf, fish were kept at a maximum density of 0.4 embryo/mL and fish medium was renewed once a day and from 9 dpf until 14 dpf twice a day. Larvae were fed twice daily with paramaecia from 4–6 dpf. From 7–9 dpf, they were fed paramecia and SDS-100 (Special Diets Services, Essex, UK) twice daily. From 10–13 dpf, they were additionally fed freshly harvested *Artemia* nauplii twice daily. The larvae of 9 and 14 dpf were not fed on the day of the experiment to limit the amount of food in the gastrointestinal system. For each developmental stage—i.e., 7, 26, 50, 74, 98, and 122 hpf and 9 and 14 dpf—three biological replicates were used in the CYP activity assays. At the end of each assay, embryos and larvae were euthanized by transferring them to a tricaine methane sulfonate (MS222, Sigma-Aldrich) solution with a final concentration of 1 mg/mL. Fish husbandry and all experiments were carried out in strict accordance with the EU Directive on the protection of animals used for scientific purposes (2010/63/EU) [89]. The animal protocols applied in this study were evaluated and approved by the Ethical Committee of Animal Experimentation from the University of Antwerp (Antwerp, Belgium) (ECD 2018–08; 05 March 2018).

#### 4.2.2. Benzyloxy-Methyl-Resorufin Assay in Zebrafish Embryos and Larvae

**Figure 7:**
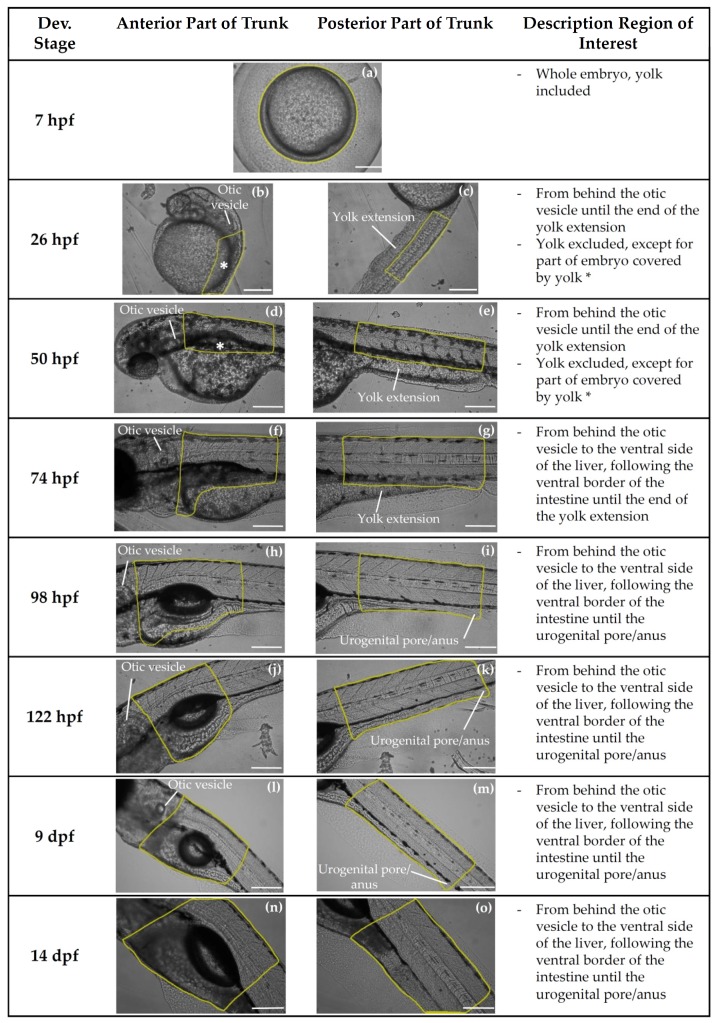
Description of region of interest used for the quantitative and qualitative analysis of resorufin formation in zebrafish embryos and larvae at 7 h post-fertilization (hpf) (**a**), 26 hpf (**b**,**c**), 50 hpf (**d**,**e**), 74 hpf (**f**,**g**), 98 hpf (**h**,**i**), 122 hpf (**j**,**k**), 9 d post-fertilization (dpf) (**l**,**m**) and 14 dpf (**n**,**o**) after exposure to benzyloxy-methyl-resorufin (BOMR) or 7-ethoxyresorufin (ER). The yellow frame indicates the region of interest in the embryo or larva. Since for most embryos/larvae the complete trunk region did not fit within one image, pictures of anterior and posterior trunk were taken separately. For the quantitative analysis of resorufin formation in each embryo/larva, average pixel intensities of anterior and posterior trunk images were combined. Figure 7 (**a**) shows a vegetal pole view of the embryo. In Figure 7 (**b–o**) lateral views of the anterior and posterior part of the trunk region are shown. Scale bar: 200 µm; (**b**,**c**): anterior top and dorsal right; (**d–o**): anterior left and dorsal top.

Zebrafish embryos and larvae of 7, 26, 50, 74, 98, and 122 hpf and 9 and 14 dpf were incubated with 4 µM BOMR (Vivid^®^ BOMR Substrate, P2865, Thermo Fisher Scientific) dissolved in fish medium. Zebrafish embryos of 26 hpf were manually dechorionated prior to incubation with the BOMR substrate. Since approximately 50% of the embryos of 50 hpf had already spontaneously hatched at this stage, manually dechorionated and spontaneously hatched embryos were used in the assay according to a 1/1 ratio. Embryos of 7 hpf were not dechorionated due to lower survival rates caused by the procedure at this stage [90]. For each developmental stage, a blank group—embryos/larvae incubated in fish medium without substrate—was included. Zebrafish embryos/larvae incubated with 1.7 µM 7-ethoxyresorufin (ER) (Resorufin ethyl ether, Sigma-Aldrich)—a CYP1-specific substrate—in fish medium were used as positive control since positive results have been described in literature [30,34]. Each embryo/larva was transferred in 150 µL of fish medium to a well of a black 96-Well Cell Imaging Plate with clear 25 µm film bottom (Eppendorf Cell Imaging Plates, 0030741013, Eppendorf, Hamburg, Germany). Subsequently, 50 µL of the substrate solution (final concentration/embryo or larva: 4 µM BOMR or 1.7 µM ER) or 50 µL of fish medium (blank) was added to the embryo/larva followed by incubation for 60 min at 28.5 °C under light-protected conditions. Following incubation, each embryo/larva was immobilized by the addition of tricaine methane sulfonate (MS222, Sigma-Aldrich) with a final concentration of 0.2 mg/mL per embryo or larva. Finally, the formation of resorufin was analyzed by means of an inverted fluorescence microscope (Olympus IX 71, Olympus Corporation, Shinjuku, Tokyo, Japan) with a 10× objective at *λ*_ex_ 510–550 nm and *λ*_em_ ≥ 570–590 nm. Grayscale images (8 bit) were acquired by means of the CellSens Software (Olympus Corporation) using a fixed gain and exposure setting for all images. A qualitative and quantitative analysis of resorufin formation was performed in the anterior and posterior trunk region of the zebrafish embryo/larva (Figure 7). The trunk region was selected for analysis as it contains the major CYP-containing organ, i.e., the liver, together with some extrahepatic organs that are involved in drug metabolism, i.e., intestine, kidney, cardiovascular system. Since the trunk has not been developed yet at 7 hpf (gastrulation period [6]), analysis of metabolite formation was accomplished in the whole embryo. For each biological replicate, at least four embryos/larvae of each condition were evaluated from which the two best positioned embryos/larvae were used for further analysis (Table 1).

#### 4.2.3. Mathematical and Statistical Analyses

Qualitative analysis of resorufin formation was performed by visual inspection of an overlay (grayscale/bright-field-overlay) image of the trunk region of the embryo/larva. Quantitative analysis of resorufin formation was performed in a grayscale image by measuring the average pixel intensity within the trunk region (Figure 7) using the ImageJ software (version 1.50i; National Institutes of Health, Bethesda, MD, USA). The corrected integrated density of resorufin was calculated by means of a formula: (average pixel intensity of region of interest—background average pixel intensity) × area of interest. The LLOD (integrated density value: 522,443) and LLOQ (integrated density value: 1,421,607) were determined based on the mean and standard deviation of the corrected integrated density for the blank embryos/larvae as described by Şengül [88]. Calculation of corrected integrated density and detection and quantification limits was performed in Microsoft Excel^®^ 2016 (Microsoft Corporation). The quantitative results from the in vivo assay with BOMR and ER that showed values above the LLOQ were statistically analyzed using IBM SPSS Statistics (version 25; IBM). Statistical analysis was performed on the following developmental stages: 74, 98, 122 hpf, 9 and 14 dpf for the BOMR assay and 7, 26, 50, 74, 98, and 122 hpf for the EROD assay. A nonparametric Levene’s test was used to test homogeneity of variances. Subsequently, the results for these age groups were subjected to a Kruskal-Wallis test, followed by pairwise comparisons (Mann-Whitney test) to detect differences between the groups. Differences were considered statistically significant when *p* ≤ 0.05.

### 4.3. mRNA Expression of Phase I and Phase II Enzymes and P–Glycoprotein

#### 4.3.1. Fish Maintenance and Breeding

The same samples used in the ontogeny study of Vergauwen et al. [54] were used here. Adult zebrafish (*Danio rerio*, in-house wild-type zebrafish line) were housed under the same environmental conditions as described for the in vivo study. For the collection of zebrafish embryos, one female and one male fish were transferred to a breeding tank and separated from each other the evening before mating. The next morning, the divider was removed when the lights turned on. Within 45 min, eggs were collected and pooled from multiple spawning groups and randomly distributed into plastic beakers with an initial density of 45 embryos per 100 mL. The density was gradually decreased to 7 larvae per 100 mL at 10 dpf with gentle aeration initiated at 9 dpf. Zebrafish embryos were raised in fish medium that had the same composition as the water in the ZebTEC housing system at 28.5 °C with 14/10 h light/dark cycle. Fish medium was renewed daily. At 15 dpf, larvae were transferred to a ZebTEC zebrafish housing system. Fish were fed twice daily with paramaecia from 4–6 dpf. From 7–9 dpf, they were fed paramecia and SDS-100 (Special Diets Services) twice daily. From 10 dpf, they were additionally fed freshly harvested *Artemia* nauplii twice daily. Starting at 15 dpf paramecia feeding was reduced to once daily. From 20 to 32 dpf, they were fed *Artemia* nauplii once daily and SDS-100 twice daily.

Embryos/larvae/juveniles were sampled at 25 time points, each time point containing four independent biological replicates (Table 2). Whole body samples were snap-frozen in liquid nitrogen and stored at −80 °C until processing. Fish husbandry and all experiments were carried out in strict accordance with the EU Directive on the protection of animals used for scientific purposes (2010/63/EU) [89]. The animal protocols applied in this study were evaluated and approved by the Ethical Committee of Animal Experimentation from the University of Antwerp (Antwerp, Belgium) (ECD 2015-51; 18 September 2015).

#### 4.3.2. Quantification of mRNA Levels by means of qPCR

For each time point, the mRNA expression of seven phase I enzymes, i.e., *CYP1A*, *CYP1B1*, *CYP1C1*, *CYP1C2*, *CYP2K6*, *CYP3A65*, and *CYP3C1*, two phase II enzymes, i.e., sulfotransferase 1st1 (*SULT1ST1*) and uridine diphosphate glucuronosyltransferase 1A1 (*UGT1A1*), and one P–glycoprotein, i.e., ATP-binding cassette b4 (*abcb4*) transporter was analyzed by means of quantitative polymerase chain reaction (qPCR). Except for *CYP2K6*, for which primers were designed in-house, all primer sequences were obtained from literature (Table 3). Most amplicons spanned two exons and the sequence of the amplicons was confirmed using the National Center for Biotechnology Information’s Basic Local Alignment Search Tool (NCBI, BLAST) [91] to verify specific sequence alignment with the targeted gene in the zebrafish genome. All primers were ordered from Eurogentec (Liège, Belgium).

RNA was extracted from homogenized whole zebrafish body samples using the NucleoSpin^®^ RNA isolation kit (Macherey-Nagel, Düren, Germany) according to the manufacturer’s protocol, including a DNAse treatment. RNA purity and integrity were confirmed using a NanoDrop spectrophotometer (NanoDrop Technologies, Rockland, DE, USA) and a BioAnalyzer (Agilent Technologies, Diegem, Belgium). All samples had minimal A_260 nm_/A_280 nm_ ratios of 2.1 and minimal RNA integrity number (RIN) of 7.9. Complement DNA (cDNA) was synthesized from the extracted RNA using a RevertAid H Minus First Strand cDNA Synthesis Kit (Thermo Fischer Scientific) according to the manufacturer’s instructions, with random hexamer primers. Subsequently, cDNA was diluted to 70 ng/µL in 0.1% diethylpyrocarbonate (DEPC)-treated water prior to its use as a template for the qPCR reaction. Quantitative PCR reactions were performed in an MX3005P instrument (Agilent Technologies) using the Brilliant II SYBR^®^ Green qPCR Master Mix (Agilent Technologies). Each qPCR reaction contained 350 ng cDNA, 10 pmol forward primer and 10 pmol reverse primer in a final volume of 19.3 µL. Thermal cycling profiles were: an initialization step of 10 min at 95 °C, followed by 40 cycles of a denaturation step of 20 s at 95 °C, an annealing step of 40 s at 55 °C (51 °C for *CYP2K6*) and an elongation step of 30 s at 72 °C. Melting curves were assessed to confirm specific amplification. Primer efficiencies were determined using duplicate standard curves with four concentrations in a 1.5-fold dilution series of a mixed cDNA sample based on different time points. The same standard curves were included in each qPCR run to correct for inter-run differences. 18S ribosomal RNA (*18S*) and beta actin 1 (*actb1*) (Table 3) were selected from five potential reference genes using geNorm [92]. Both reference genes were used in further analysis of the qPCR data.

#### 4.3.3. Mathematical and Statistical Analyses.

The transcript abundance of each sample was divided by the geometric mean of *18S* and *actb1* transcript abundances in that sample to normalize the experimental data for reference gene expression [92]. An inter-run calibration was performed using qbase^+^ software (version 3.1; Biogazelle, Zwijnaarde, Belgium). For each gene, the resulting data were divided by the average abundance at the time point with the lowest expression for that gene and subsequently log2 transformed to increase the resolution. The log2 relative quantities were analyzed using R Statistical Software (version 3.4.3; RStudio Inc., Boston, MA, USA). The R code as previously published by Vergauwen et al. (Supplementary Data in [54]) was used in the analyses. The aim of the statistical approach was to determine when mRNA expression data at particular time points significantly deviate from trends in the data, thereby defining critical points (e.g., local maxima and minima) of mRNA expression. In brief, local weighted regression (lowess) along with residual plots were used to identify possible outliers in each dataset. Next, local regression (loess) with the simplest fit span was utilized to estimate the non-linear trends in responses for each gene. Selection of the loess model was verified by confirming that the residuals had no pattern over time. Critical points (i.e., minima, maxima, and inflection points) in the data were determined when the derivative of the best-fit function through the data equals 0. Finally, to obtain confidence intervals around each critical point, bootstrapping techniques were used to find estimates of the slope of the responses.

## 5. Conclusions

The extensive use of zebrafish embryos as an alternative animal model in developmental toxicity studies increases the demand for a detailed investigation of their intrinsic biotransformation capacity since the embryos cannot rely on maternal metabolism of the xenobiotics. The present study contributes to a better understanding of the ontogeny of metabolism and transport of xenobiotics in the zebrafish, and suggests that, in general, the disposition of xenobiotics in zebrafish embryos is immature during a major part of the organogenesis period, i.e., before 72 hpf. This may lead to false negative results in the case of proteratogens, whereas the teratogenic potential might increase in the case of teratogens since immature biotransformation might result in a higher internal concentration of the teratogenic parent compound. Full capacity appears to be reached by the end of organogenesis (i.e., 120 hpf), although *CYP1*—except *CYP1A*—and *SULT1ST1* showed to be already mature in early embryonic development. Furthermore, the present study showed that in vitro CYP activity assays with microsomes prepared from whole zebrafish organisms do not always reflect the in vivo activity and can underestimate the biotransformation capacity of the organisms.

In literature, CYP activity and expression studies mainly focus on zebrafish embryonic development, whereas in the present study the experimental time window has been extended to the beginning of the juvenile period. The study showed that CYP activity and expression mainly remained stable during the larval period. However, regarding the phase II enzymes and P-glycoprotein, activity studies need to be performed to draw conclusions on their role in drug metabolism during zebrafish development.

## Figures and Tables

**Table 1:** Comparison of the experimental setup between in vitro and in vivo study.

Experimental Setup	In Vitro	In Vivo
Developmental stage	5, 24, 48, 72, 96, 120 hpf ^1^ 9 and 14 dpf Adults	7, 26, 50, 74, 98, 122 hpf 9 and 14 dpf
Samples	Microsomes from whole embryos/larvae/adults	Intact embryos and larvae
Substrate CYP activity	BOMR	BOMR
Negative control/Blank	Supersomes	Embryos/larvae in fish medium
Positive control	Adult zebrafish liver microsomes ^1^	Embryos/larvae incubated with ER ^2^
Biological replicates	Three (5–120 hpf; 14 dpf; adults) Two (9 dpf)	Three/developmental stage
Technical replicates	Two for 5–120 hpf Six for 9 and 14 dpf Three for adults	Two
Detection of resorufin formation	Microplate reader	Fluorescence microscope

^1^ Verbueken et al., 2017 [25]; ^2^ Otte et al., 2010 [34]. Hpf, h post-fertilization; dpf, d post-fertilization; CYP, cytochrome P450; BOMR, benzyloxy-methyl-resorufin; ER, 7-ethoxyresorufin.

**Table 2:** Overview of sampling time points for analysis of mRNA expression.

Time Point	Hpf	Dpf	Number of Organisms/Biological Replicate
1	1.5	0.06	30
2	6	0.25	30
3	14	0.58	30
4	24	1	20
5	36	1.5	20
6	48	2	20
7	60	2.5	20
8	72	3	20
9	84	3.5	20
10	96	4	10
11	120	5	10
12	144	6	10
13	192	8	10
14	240	10	10
15	288	12	10
16	336	14	10
17	384	16	10
18	432	18	10
19	480	20	10
20	528	22	10
21	576	24	10
22	624	26	10
23	672	28	10
24	720	30	10
25	768	32	10

Hpf, h post-fertilization; dpf, d post-fertilization.

**Table 3:** Primer sequences of zebrafish target genes and reference genes used for quantitative polymerase chain reaction analyses.

Gene	Sequence (5′ to 3′)	Accession Number	Reference
**Target Genes**			
*CYP1A*	FW: GCATTACGATACGTTCGATAAGGAC RV: GCTCCGAATAGGTCATTGACGAT	NM_131879.1	Goldstone et al., 2010 [22]
*CYP1B1*	FW: GAGCACCGAAAGACCATTTCA RV: ATGGTCGGTGGCACAAACTC	NM_001045256.1 NM_001145708.1	Olsvik et al., 2014 [93]
*CYP1C1*	FW: AGTGGCACAGTCTACTTTGAGAG RV: TCGTCCATCAGCACTCAG	NM_001020610.2	Goldstone et al., 2010 [22]
*CYP1C2*	FW: GTGGTGGAGCACAGACTAAG RV: TTCAGTATGAGCCTCAGTCAAAC	NM_001114849.1	Jönsson et al., 2007 [72]
*CYP2K6*	FW: CCAGCTTTGTCCCTGTTTCTT RV: GCAGAGAGTTCAGCCTGTGAT	NM_200509.2	Designed in-house
*CYP3A65*	FW: CTTCGGCACCATGCTGAGAT RV: AGATACCCCAGATCCGTCCATA	NM_001037438.1	Chang et al., 2013 [94]
*CYP3C1*	FW: TCCAGACCTCTGGGAGTCTCCTAAT RV: GCATGAAGGCACACTGGTTGATCT	NM_212673.1	Shaya et al., 2014 [61]
*SULT1ST1*	FW: GTTCCTTCTTGGGTTTGTCT RV: CTGGCAGAGTGGAATAGTTG	NM_182941.1	Liu et al., 2011 [95]
*UGT1A1*	FW: TCCTTTGCCGCAGCATGTAT RV: ACTCTCTGGCTTTGGCTTCG	NM_001037428.2	Wang et al., 2014 [96]
*abcb4*	FW: TACTGATGATGCTTGGCTTAATC RV: TCTCTGGAAAGGTGAAGTTAGG	NM_001316714.1 NM_001114583.2	Fischer et al., 2013 [53]
**Reference Genes**			
*18S*	FW: CGGAGAGGGAGCCTGAGAA RV: AGTCGGGAGTGGGTAATTTGC		Biga et al., 2005 [97]
*actb1*	FW: AAGTGCGACGTGGACA RV: GTTTAGGTTGGTCGTTCGTTTGA	NM_131031	Gonzalez et al., 2006 [98]
*hprt1*	FW: GTGGCTCTATGTGTGCT RV: CCTCCACAATCAAGACG	NM_212986.1	Bio- Engineering Com.(Shanghai, China)
*rpn2*	FW: TTGAGTTCAGCCAGCGT RV: TGGCAACAAATCGGCG	NM_212748.1	De Wit et al., 2008 [99]
*ef1a*	FW: TGTCCTCAAGCCTGGTAT RV: CATTACCACGACGGATGT	NM_131263	Houbrechts et al., 2016 [100]

FW, forward primer; RV, reverse primer; CYP, cytochrome P450; sult, sulfotransferase; UGT, uridine diphosphate glucuronosyltransferase; abc, ATP-binding cassette; 18S, 18S ribosomal RNA; actb1, beta actin 1; hprt1, hypoxanthine phosphoribosyltransferase1; rpn2, ribophorin 2; ef1a, eukaryotic translation elongation factor 1 alpha 1.

## Abbreviations

ABC	ATP-binding cassette
AhR	Aryl hydrocarbon receptor
BOMR	Benzyloxy-methyl-resorufin
CRO	Contract research organization
CYP	Cytochrome P450
Dpf	Days post-fertilization
ER	7-Ethoxyresorufin
EROD	Ethoxyresorufin-*o*-deethylase
FET	Fish embryo acute toxicity test
FMO	Flavin monooxygenase
Hpf	Hours post-fertilization
LLOD	Lower limit of detection
LLOQ	Lower limit of quantification
MP	Microsomal protein
MXR	Multixenobiotic resistance
OECD	Organization for Economic Co-operation and Development
PXR	Pregnane X receptor
qPCR	Quantitative polymerase chain reaction
RA	Retinoic acid
S.B.	Swim bladder
SULT	Sulfotransferase
TCDD	2,3,7,8-Tetrachlorodibenzo-*p*-dioxin
UGT	Uridine diphosphate glucuronosyltransferase
ZEDTA	Zebrafish embryo developmental toxicity assay
ZEM	Microsomes prepared from whole zebrafish embryo homogenates
ZLaM	Microsomes prepared from whole zebrafish larva homogenates
ZLM	Zebrafish liver microsomes
ZM	Microsomes prepared from whole adult zebrafish homogenates
